# Host immune responses to chlamydial inclusion membrane proteins B and C in Chlamydia trachomatis infected women with or without fertility disorders

**DOI:** 10.1186/1477-7827-7-38

**Published:** 2009-04-28

**Authors:** Rishein Gupta, Pragya Srivastava, Harsh Vardhan, Sudha Salhan, Aruna Mittal

**Affiliations:** 1 Institute of Pathology-ICMR, Safdarjang Hospital Campus, Post Box no. 4909, New Delhi-110 029, India; 2 Department of Gynaecology & Obstetrics, Safdarjung Hospital, New Delhi-110 029, India

## Abstract

**Background:**

With an increase in the number of putative inclusion membrane proteins (incs) in chlamydial genomes, there is a need for understanding their contribution in host-pathogen interactions. Thus in this study we determined the host mucosal and peripheral immune responses to incs (IncB and IncC) of Chlamydia trachomatis (CT).

**Methods:**

Female patients (n = 296) attending the gynaecology out patient department of Safdarjung hospital, New Delhi were enrolled for the study and were clinically characterized into two groups; CT-positive fertile women (n = 38) and CT-positive women with fertility disorders (n = 29). Uninfected healthy fertile women were enrolled as controls (n = 31). Gene specific PCRs were used for detection of incB and incC genes in endocervical samples of CT-positive patients. ELISA and Western blot assay were used for detection of IgA and IgG antibodies to IncB and IncC in cervical washes and sera. Effect of IncB and IncC stimulation of cervical cells and PBMCs on cellular proliferation and cytotoxity was determined using MTT assay and Lactate dehydrogenase (LDH)-cytotoxicity assay respectively. Modulation of cytokines (Interleukin (IL)-1 Beta, IL-4, IL-5, IL-6, IL-10, Interferon-gamma, IL-12, Tumor Necrosis Factor-alpha and Granulocyte macrophage colony-stimulating factor (GM-CSF)) in cervical cells and PBMCs upon stimulation with IncB and IncC was determined by real-time reverse-transcriptase (RT)-PCR and ELISA. Further, CD4 positive T cells were purified from cervical cells and peripheral blood mononuclear cells (PBMCs) and secreted cytokines (Interferon-gamma and IL-4) were evaluated by ELISPOT and real-time RT-PCR.

**Results:**

Using MTT assay, significantly high proliferative responses (P < 0.05) were observed in inc-stimulated cervical cells and PBMCs from CT-positive fertile women compared to CT-positive women with fertility disorders and controls. Interferon-gamma, IL-12 and GM-CSF were found to be elevated in inc-stimulated cervical cells and PBMCs of CT-positive fertile women compared to CT-positive women with fertility disorders and controls (P < 0.05). In contrast, IL-1 Beta, IL-4, IL-5, IL-6 and IL-10 levels were found to be higher in CT-positive women with fertility disorders compared to CT-positive fertile women and controls (P < 0.05). Interferon-gamma secreting cells and mRNA expression in inc-stimulated cervical and peripheral CD4 positive T cells were significantly higher (P < 0.05) in CT positive fertile women compared to CT-positive women with fertility disorders.

**Conclusion:**

Our data overall suggests that CT incs, IncB and IncC modulate host immune responses and may have a role in protection/pathogenesis of genital chlamydial infection in women.

## Background

Infection with *Chlamydia trachomatis *(CT), a sexually transmitted pathogen leads to the development of debilitating diseases such as chronic pelvic pain, pelvic inflammatory disease (PID), infertility and ectopic pregnancy in women [1-3]. With approximately 90 million new cases being reported each year, chlamydial infections result in substantial morbidity and socioeconomic burden worldwide [2,4].

Chlamydial infection and propagation within host genital mucosal epithelial cells rely upon a unique biphasic developmental cycle inside a membrane-bound vacuole termed inclusion. The chlamydial life cycle consists of two morphological forms, an extracellular, infectious form – the elementary body (EB) which enters host cells and differentiates into an intracellular, metabolically-active reticulate body (RB). Vesicles of the endo- and exocytic pathways and proteins involved in vesicle trafficking are recruited to the inclusion to facilitate effective chlamydial infection in infected cells [5-8]. Chlamydial proteins called inclusion membrane proteins (incs) translocated to the inclusion membrane (IM) by a Type III secretion system are potentially involved in mediating such vesicular trafficking processes [9,10]. Host cell components are capable of interacting and modifying segments of incs which are exposed to the cytosolic face of the inclusion [11,12]. Further, incs have been reported to generate humoral immunity in infected humans and animals [13-17] and cellular immunity by eliciting MHC class I-restricted CD8^+ ^T cell responses [18-20]. Studies on the involvement of CT IncA in homotypic membrane fusion via N-terminal SNARE-like motifs [8,21] and IncA mutant stains have been instrumental in elucidating the role of incs in disease pathogenesis and inducing modified host-cell transcriptional responses [22]. CT IncB and IncC with homologues in *C. pneumoniae *[23], *C. psittaci *[16], *C. muridarum *[24] and *C. abortus *[25] may be involved in processes like inclusion formation, transportation to perinuclear space and evasion of early lysosomal fusion as their corresponding genes are expressed within 0.5 hours of the infection cycle and coincides with these events.

Emerging evidence on the increasing number of putative incs in all chlamydial genomes [14,23-31] has highlighted the importance of this protein family as probable candidates for intervention of chlamydial disease. Our knowledge on structure and functions of incs has been limited due to the absence of a system for genetically transforming chlamydiae. Alternate strategies like microinjection studies that reported inc phosphorylation by host cell protein phosphokinases [11,12] and yeast two hybrid systems showing IncG interaction with host cell protein (14-3-3β) [12], have put forward the hypothesis that places incs at a central point in the interaction between infected host cells and the chlamydial developmental forms. There is however, no evidence on the role of incs and host immunity in terms of clearance or establishment of infection in the genital tract. We have recently reported seroprevalence of antibodies to chlamydial incs in CT-positive women [32]. Further Inc antibody levels were found to be positively correlated with CT MOMP antibodies and were found to be higher in CT-positive cervicitis patients. These findings brought forward a pondering question- Are chlamydial incs involved in host cell-mediated immune responses and do they have a role to play in pathogenesis of chlamydial infection? This prompted us to further assess cellular immune responses to IncB and IncC in CT-infected women with or without fertility disorders and controls.

In this study gene-specific PCRs were used for detection of *incB *and *incC *genes in endocervical samples. Cellular proliferation using MTT assay and cellular damage using LDH assay were assayed in IncB or IncC stimulated cervical cells and peripheral blood mononuclear cells (PBMCs) in enrolled women. Effect of IncB and IncC on cytokine production upon stimulation of cervical cells and PBMC was evaluated by cytokine specific real-time reverse-transcriptase (RT)-PCR assay, ELISA and ELISPOT.

## Methods

### Study population

After obtaining informed written consent, 296 patients attending the gynaecology outpatient department, Safdarjung Hospital, New Delhi, India were enrolled for the study. Thirty-six healthy age-matched controls attending the family planning department for birth control measures were also enrolled. The study received approval from the hospital's ethics review committee. Procedures followed for sample collection were in accordance with the ethical standards for human experimentation established by the Declaration of Helsinki of 1975 (revised in 1983). At recruitment, a detailed clinical questionnaire was administered to each patient for collecting information on reasons for referral, gynaecology history including menstruation, symptoms of genital and urinary tract infection, obstetric and medical histories. Patients with positive urine pregnancy test, recent antibiotic therapy and with genital tuberculosis were excluded from the study.

Fertile women were those having their last child birth within the last 4 months to 1 year and testing positive for CT during last pregnancy. Women with fertility related disorders included women with infertility and multiple spontaneous abortions. Infertile women were identified as those, which lack recognized conception after 1.5 to 2 years of regular intercourse without the use of contraception. The infertile group included women with referred diagnostic laparoscopy [33]. Patients with endometriosis and male factor related infertility were not included in this group. Women with multiple spontaneous abortions (more than 2) were described as those having delivery of pre-viable foetus before the 20^th ^week of gestation.

Since variations in sex hormones are known to influence cytokines concentrations and immune cell population, cervical samples were collected during mid-cycle (median 13 days, range 11^th ^to 15^th ^day of the menstrual cycle). None of the patients had sexual intercourse 3 days or more prior to collection of samples.

### Collection of samples

The vulva was examined for lesions and the cervix for warts, ulcers, ectopy, erythma and discharge, if any. After cleaning the endocervix with a cotton swab (Hi Media, Mumbai, India), endocervical swabs were collected from patients for diagnosis of CT and other STD pathogens. For collection of cervical cells, a cytobrush was placed within the endocervical canal so that the cells from the endocervical region and the zone between the endocervical and ectocervical regions (transformation zone) could be obtained. The cytobrush was then transferred to a sterile centrifuge tube containing sterile phosphate-buffered saline (PBS) (pH 7.2) supplemented with 100 U penicillin/ml, 100 μg streptomycin/ml and 100 μg glutamine/ml. No samples were collected from patients with friable cervix and contact bleeding to ensure collection of cervical lymphocytes only. 5 ml of heparinized blood (for isolation of PBMCs) and 2 ml non-heparinized blood (for separating serum) was also collected. Samples were then stored at 4°C, transported to the laboratory and processed within 1 h.

### Laboratory diagnosis

Endocervical samples were tested for chlamydial positivity by PCR analysis using CT specific 200 base pair (bp) plasmid primers [34]. Diagnosis for other STD pathogens was done as described earlier [33]. In brief, *Neisseria gonorrhoeae*, *Mycoplasma hominis *and *Ureaplasma urealyticum *were detected by culture method along with. In addition *Candida sp*., bacterial vaginosis and *Trichomonas vaginalis *were diagnosed by microscopy on gram stained smears.

### CT *incB *and *incC *PCR

Two sets of primers, set 1(S1) and set 2(S2) were used for PCR amplification of both CT *incB *(CT 232) and *incC *(CT 233) genes in all endocervical samples. For PCRs, reaction volumes (total volume 25 μl) contained: 5 μl DNA, 2.5 μl 10× PCR buffer (200 mM Tris-HCl (pH 8.4), 500 mM KCl), 0.5 μl 10 mM dNTP mixture, 0.75 μl 50 mM MgCl_2_, 3 μl of each primer (5 pM/μl), and 0.1 μl Taq DNA Polymerase (5 U/μl, Invitrogen, USA). For *incB*- and *incC*-S1 primer sets were same as mentioned earlier [35]. PCR reaction tubes were subjected to cycling parameters for *incB-*S1 amplification at 94°C/1 min, 55°C/45 sec, 74°C/1 min for 34 cycles and *incC-*S1 amplification atPCR at 94°C/1 min, 54°C/45 sec, 74°C/2 min for 36 cycles. Further, for this study, unique primer pairs (S2) for both *incB *and *incC *genes were designed using Primer3 software (primer3_www.cgi v 0.1 beta 1a). Primer sequences for *incB *S2 were: forward primer 5'-GGA TCT ATG GTT CAT TCT GTA TAC AAT TC-3' and reverse primer 5'-GCA TGT CCT ATT CTT GAG GTT TTG TTT G-3'; for *incC *S2 forward primer 5'-CGG GGT ACC ATG ACG TAC TCT ATA TCC GAT-3' and reverse primer 5'-CCC AAG CTT TTA GCT TAC ATA TAA AGT TTG AG-3'. For S2 primer sets, all reaction tubes were subjected to cycling parameters for *incB-*S2 amplification at 94°C/1 min, 57°C/50 sec, 74°C/2 min for 35 cycles and *incC-*S2 amplification atPCR at 94°C/1 min, 61°C/45 sec, 74°C/2 min for 32 cycles.

All primers were commercially synthesized and HPLC purified (MWG-Biotech AG, Ebersberg, Germany). All PCR reactions were performed on DNA Eppendorf Mastercycler personal Thermal Cycler (Eppendorf GmbH, Hamburg, Germany). Amplification products were electrophoresed on 1.2% (w/v) agarose gel and ethidium bromide (0.5 pg/ml) stained gels were visualized on Alpha Imager gel documentation system (AlphaInnotech, San Leandro, USA).

### Expression of CT IncB and IncC proteins

Recombinant clones containing full length gene sequences of *incB *and *incC *cloned into pGEX expression vectors (Amersham Pharmacia Biotech Inc., NJ, USA) were obtained as a kind gift from Dr. Guangming Zhong at Department of Microbiology and Immunology, University of Texas Health Science Center at San Antonio, USA. These clones were further propagated in Terrific Broth (Amresco, Ohio, USA) and production of glutathione S-transferase (GST) fusion proteins was performed as described elsewhere [36]. Briefly, production of GST fusion proteins was induced with isopropyl-D-thiogalactopyranoside (IPTG; Invitrogen, CA, USA) and GST fusion proteins were extracted by lysing the bacteria via sonication in a Triton X-100 lysis buffer (1% Triton X-100,1 mM phenyl methyl sulfonyl fluoride, 75 U of aprotinin/ml, 20 M leupeptin, and 1.6 M pepstatin) (Amresco). After a high speed centrifugation to remove debris, the fusion protein-containing supernatants were further purified with glutathione-conjugated agarose beads (Amersham) according to manufacturer's instructions. The protein concentration was determined with the Bradford assay (Sigma-Aldrich, MO, USA). The non-specific effects of LPS contamination were controlled by treating recombinant proteins with polymyxin B (Sigma-Aldrich). The fusion proteins were checked on sodium dodecyl sulphate (SDS)-polyacrylamide gels stained with a Coomassie blue dye (Sigma-Aldrich). Bacterial lysate samples that showed a prominent band at the expected molecular weight position were separated into aliquots and frozen at -80°C to be used in further assays.

### Detection of antibodies against CT IncB and IncC

CT IncB and IncC specific IgG and IgA titres in cervical washes and sera were determined by ELISA as previously described [37]. Briefly, 96-well plates were coated with 1 μg antigen/well and 100 μL of cervical washes or sera from patients were added per well in serial dilutions. Free GST was coated in separate wells to serve as negative controls. After incubation for 2 h at 37°C and subsequent washing with PBS-Tween 20 (PBS-T), plates were incubated with horse radish peroxidase (HRP)- conjugated rabbit anti- human IgG (1:10,000 dilutions) and IgA (1:5000 dilutions) antibodies (Bangalore Genei, Bangalore, India). The binding was measured at 492 nm in an ELISA reader using OPD (o-phenylenediamine dihydrochloride) as the substrate. Positive samples were defined as those yielding an absorbance (OD) value at least two standard deviations (SDs) above the mean value obtained from the panel of samples taken from the negative subjects.

CT IncB and IncC specific IgG in cervical washes and sera were further determined by Western blot assay. Inc concentrations in bacterial lysate samples were determined by Bradford assay (Bio-Rad Laboratories, CA, USA) with bovine serum albumin as standard. 2 μg Incs were electrophoresed on 12% (SDS)-polyacrylamide gels, transferred to polyvinyl difluoride (PVDF) membrane (Bio-Rad) and reversibly stained with Ponceau S (Sigma-Aldrich) to confirm complete transfer. Membranes were then blocked with 5% non-fat dry milk in PBS-T and strips of membranes were incubated with individual patients' cervical washes or sera (1:100). Membranes were further incubated with HRP-conjugated rabbit anti- human IgG (1:1000 dilutions) antibodies and developed by Diaminobenzamide (DAB) (Sigma-Aldrich) as a detection system. Images were analyzed with the help of ImageJ software .

### Cell cultures

PBMCs were prepared by Ficoll-Hypaque density gradient centrifugation (Sigma-Aldrich) whereas cervical cells were isolated and counted as described earlier [38]. Cells were then washed three times with Hank's balanced salt solution (Sigma-Aldrich) and suspended in RPMI-1640 medium (Sigma-Aldrich) supplemented with 10% heat-inactivated human AB serum. Subsequently the cells were then cultured in triplicate (0.6 × 10^5 ^cells/well) in round-bottomed 96-well plates with or without stimulants in a total volume of 200 μl and were incubated in humidified 5% CO_2 _at 37°C for 12 h (for mRNA expression by real-time RT-PCR) and 72 h (for cell proliferation assays, ELISA and cell proliferation assays).

### Stimulants

Cells were stimulated with/without CT serovar D (a human clinical genital isolate) whole EBs at multiplicity of infection 2 (to serve as CT positive control), IncB (1 μg/ml) and IncC (1 μg/ml). Phytohaemaglutinin (PHA 2 μg/ml) (Sigma-Aldrich) and free GST were used as positive control mitogen and negative control respectively in each experiment. CT serovar D was grown on confluent McCoy cell monolayers as described previously [39] and EBs were harvested and purified on Renograffin gradients. Optimum concentrations of antigens and mitogen were determined in preliminary experiments as minimum concentrations giving maximal proliferation at different time intervals post stimulation.

### Cell proliferation assay

A colorimetric assay based on MTT (3-(4, 5-dimethylthiazol-2-yl)-2, 5- diphenyl tetrazolium bromide) (Sigma-Aldrich) was performed to measure the proliferative activity of cervical cells and PBMCs upon stimulation with incs. In brief, at 72 h post stimulation, 10 μl of 5 mg/ml MTT solution was added to cell cultures, which was incubated for 3 h at 37°C. The MTT reaction was terminated by the addition of acidic isopropanol (40 mM HCl). Absorbance was taken at 570/650 nm using a μQuant microplate spectrophotometer (BioTek Instruments, Winooski, VT). The MTT results were expressed as stimulation indices (SI) and an SI value of 2 or more was considered a positive response.

### Cell cytotoxity assay

A colorimetric assay based on measurement of lactate dehydrogenase (LDH) release was performed to measure the cytotoxicity of cervical cells and PBMCs upon stimulation with incs. Release of LDH from damaged cells was measured using LDH-Cytotoxicity Assay Kit (BioVision Research Products,  California, USA) according to manufacturer's instructions. Absorbance was taken at 500/600 nm (for LDH) using a μQuant microplate spectrophotometer (BioTek Instruments, Winooski, VT). For LDH, positive samples were those yielding an absorbance (OD) value > mean ± 2SD than that of controls.

### RNA extraction and real-time RT-PCR analysis for cytokines

Total RNA from stimulated cervical cells and PBMCs was isolated using RNeasyMini Kit (Qiagen, CA, USA), in accordance with the manufacturer's instructions and stored at -70°C. Complementary DNA (cDNA) was prepared using a SuperScript™ First-Strand Reverse Transcriptase kit (Invitrogen), in accordance with the manufacturer's instructions. The cDNA solution was diluted to 150 μl and stored at -20°C. All samples were reverse transcribed in a single batch and were all analysed for a given primer set in the same PCR run. The PCR amplification employed reagents supplied in a DyNAmo™ SYBR^® ^Green qPCR Kit (Finnzymes, Espoo, Finland), and each reaction volume (20 μl total) contained 5 μl of cDNA, and 0.5 μM of both primers. Sequences for endogenous control (β-actin) and cytokine genes (IL-1Beta (1β), IL-4, IL-5, IL-6, IL-10, IL-12, Tumor Necrosis Factor-alpha (TNF-α), Interferon-gamma (IFN-γ) and GM-CSF) used in this study were same as reported earlier by Jasper *et al*. [40]. The negative control included in each reaction consisted of H_2_O substituted for cDNA. PCR amplification was performed in an Applied Biosystems 7000 Real-Time PCR System (Applied Biosystems, California, USA) under universal cycling parameters for relative quantification of cytokine expression in target samples according to the manufacturer's instructions (Applied Biosystems User Bulletin #2: Relative Quantitation of Gene Expression). For data analysis, the 2^-ΔΔCt ^method was used to calculate fold change [41]. β-actin expression was used as a reference gene for normalization of threshold cycles (Ct).

### Quantification of secreted cytokines

Quantification of IL-1β, IL-4, IL-5, IL-6, IL-10, IL-12, IFN-γ, TNF-α and GM-CSF in culture supernatants of stimulated cervical cells and PBMCs was performed by commercially available ELISA kits (eBiosciences, San Diego, USA), in accordance with the manufacturer's instructions. The absorbance was read at 450 nm, log-log standard curves were generated and unknowns were interpolated. The minimum detectable cytokine concentrations for these assays were-IL-1β (4 pg/ml), IL-4 (2 pg/ml), IL-5 (4 pg/ml), IL-6 (2 pg/ml), IL-10 (2 pg/ml), IL-12 (4 pg/ml), IFN-γ (4 pg/ml), TNF-α (4 pg/ml) and GM-CSF (2.5 pg/ml).

### Antigen specific CD4^+ ^T cell responses

For purification of CD4^+ ^T cells, CD8^+ ^T cells were positively selected from cervical cells and PBMCs using CD8 MACS MicroBeads^® ^(Miltenyi Biotec, CA, USA) according to manufacturer's instructions. In brief, CD8^+^cells were magnetically labelled with CD8 microbeads and the cell suspension was loaded onto a MACS^® ^column which was then placed in the magnetic field of a MACS separator. The magnetically labelled CD8^+ ^T cells were retained in the column while the unlabelled cells which passed through the column were collected; this cell fraction was thus CD8 depleted and contained CD4^+ ^T cells including antigen presenting cells. This cell fraction was used for further assays and termed as CD4^+ ^T cells in the rest of the manuscript. These cells were further gently pelleted and suspended in RPMI-1640 medium (Sigma-Aldrich) supplemented with 10% heat-inactivated human AB serum. CD4^+ ^T cell cultures (7.5 × 10^4 ^cells/well) were then stimulated with IncB or IncC and incubated in humidified 5% CO_2 _at 37°C for 12 h (for RT-PCR) and 18 h (for ELISPOT). The numbers of IL-4 and IFN-γ producing cells were measured by ELISPOT assay (Diaclone, Cedex, France) as per the manufacturer's instructions and the number of cytokine-secreting cells were counted on a Bioreader^® ^4000 ELISpot Reader (Biosys, Leeds, UK). To detect transcripts at mRNA levels, real-time RT-PCR analysis of cytokines (IL-4 and IFN-γ) was performed as described above.

### Statistical analysis

The Kruskal-Wallis non parametric test was used to compare continuous variables among multiple groups. The Mann-Whitney U test was used for comparing two groups. Categorical variables were compared using χ^2 ^test. The results were presented with 95% confidence interval (CI) and P < 0.05 was considered significant. All statistical analyses were performed with Graphpad Prism Version 5 (La Jolla, CA, USA).

## Results

### Study population

**Figure 1:**
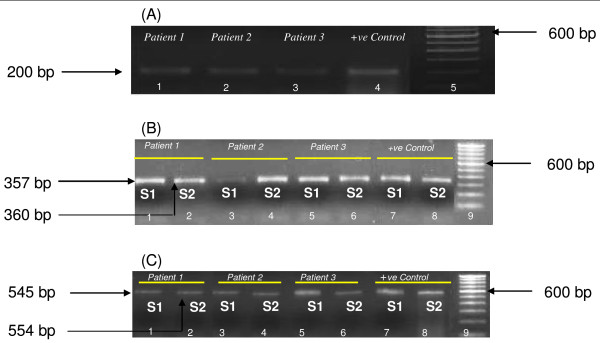
**PCR detection of *incB *and *incC *genes in endocervical samples of CT-positive patients**. (A) Gel showing 200 bp amplicons of CT plasmid. Lanes 1, 2, 3 – PCR positivity in patients 1, 2, 3 respectively; lane 4- positive control; lane 5- 100 bp Ladder (Invitrogen). (B) Agarose gel showing PCR positivity for CT-*incB *gene. Lanes 1, 3, 5- *incB *S1 PCR amplicons in patients 1, 2, 3 respectively; lane 7-positive control; lanes 2, 4, 6 – *incB *S2 PCR amplicons in patients 1, 2, 3 respectively; lane 8-positive control; lane 9 is 100 bp Ladder (Invitrogen). (C) Agarose gel showing PCR positivity for CT-*incC *gene. Lanes 1, 3, 5-*incC *S1 PCR amplicons in patients 1, 2, 3 respectively; lane 7-positive control; lanes 2, 4, 6 – *incC *S2 PCR amplicons in patients 1, 2, 3 respectively; lane 8-positive control; lane 9 is 100 bp Ladder (Invitrogen).

Cervical CT infection was diagnosed by PCR in 89 patients (Figure 1). Fifteen of these patients were found to be co-infected either with *Candida sp*., bacterial vaginosis, *T. vaginalis*, *M. hominis*, *U. urealyticum *or *N. gonorrhoeae *in the cervix and were thus excluded from the study. Seven CT-positive patients and two controls were excluded as the count of cervical cells was less than 2 million cells. All healthy controls tested negative for a current CT infection as revealed by absence of CT IgM and IgG antibodies in their sera. Three healthy controls, which were positive for CT IgG antibodies with no current chlamydial infection, were also excluded from the study. Based on clinical history and diagnosis, the patients were categorized into three groups. Group I (GI, n = 31) comprised of CT-uninfected healthy fertile controls, Group II (GII, n = 38) comprised of fertile CT-positive women and Group III (GIII, n = 29) comprised of CT-positive women with fertility related disorders. The median ages of women of GI, GII and GIII were comparable (24, 26 and 27 years, respectively).

### Detection of *incB *and *incC *genes

Detection of *incB *and *incC *in endocervical samples was done in 89 patients positive for CT infection. Figure 1 shows representative agarose gels of amplified *incB*,*incC *genes in endocervical samples by two sets of primers (S1 and S2). Visualization of 357 bp (S1) and 360 bp (S2) amplicons for *incB *gene and 545 bp (S1) and 554 bp (S2) amplicons for *incC *gene in 1.2% (w/v) agarose gel was considered as positive. All CT-positive patients showed positivity for *incB *and *incC *genes.

### Detection of antibodies against IncB and IncC by ELISA

IgG and IgA antibodies to both IncB and IncC were detected significantly high (P < 0·05) in cervical washes of CT-positive patients with or without fertility disorders compared to controls. However, there was no significant difference (P > 0.05) between IgG and IgA antibodies to both IncB and IncC in sera obtained from CT-positive women with or without fertility disorders (Table 1). Further, significant correlation was observed between antibodies to incs in cervical washes and sera in CT-positive women with or without fertility related disorders (Table 2).

**Table 1:** Prevalence of IncB and IncC specific antibodies in study population

**Groups**	**Age**	**IncB IgG^+ve^**		**IncB IgA^+ve^**		**IncC IgG^+ve^**		**IncC IgA^+ve^**	
	**Median** **(Range)**	**Serum**	**Cervical Washes**	**Serum**	**Cervical Washes**	**Serum**	**Cervical Washes**	**Serum**	**Cervical Washes**
	**n (%)**								
**Group I** **(n = 31)**	24 (21–28)	2(6)	1(3)	1(3)	1(3)	2(6)	1(3)	1(3)	1(3)
**Group II** **(n = 38)**	26 (22–29)	36(95)^a^	24(63)^b^	23(61)^c^	29(77)^d^	34(89)^e^	27(71)^f^	26(68)^g^	32(84)^h^
**Group III** **(n = 29)**	27 (22–31)	27(93)^i^	11(38)^j^	17(59)^k^	15(52)^l^	26(90)^m^	13(45)^n^	28(96)°	17(59)^p^

Note values in parenthesis represent corresponding percentages unless otherwise stated.

^a ^P = NS as compared to GIII; ^b ^P = 0.0428 as compared to GIII; ^c ^P = NS as compared to GIII;^d ^P = 0.0378 as compared to GIII; ^e ^P = NS as compared to GIII; ^f ^P = 0.0320 as compared to GIII, ^g ^P = NS as compared to GIII; ^h ^P = 0.0378 as compared to GIII; ^i, j, k, l, m, n, o, p ^P < 0.0001 as compared to corresponding GI where, Group I (GI) comprised of healthy fertile women with no CT infection, Group II (GII) comprised of CT-positive fertile women, Group III (GIII) comprised of CT-positive women with fertility disorders.

IncB- CT Inclusion membrane protein B; IncC- CT Inclusion membrane protein C; CT- *Chlamydia trachomatis*; IgG- Immunoglobulin G; IgA- Immunoglobulin A; +ve- positive. All categorical variables were compared using the χ^2 ^test.

**Table 2:** Correlation of IncB and IncC specific antibodies in study population

**Antibodies**	**GII** **r (P)**	**GIII** **r (P)**
**IncB IgG and IncC IgG in sera**	0.47 (0.0047)	0.54 (0.0148)
**IncB IgG and IncC IgG in cervical washes**	0.57 (0.0040)	0.94 (<0.0001)
**IncB IgA and IncC IgA in sera**	0.56 (0.0049)	0.92 (0.0003)
**IncB IgA and IncC IgA in cervical washes**	0.43 (0.0172)	0.60 (0.018)

Note. Correlation was tested with Spearman's correlation coefficient (r). Values in parenthesis represent corresponding significant levels unless otherwise stated.

n- number of patients, r- Spearman's correlation coefficient.

Significant correlations was observed in GII and GIII where, Group II (GII) comprised of CT-positive fertile women, Group III (GIII) comprised of CT-positive women with fertility disorders.

**Figure 2:**
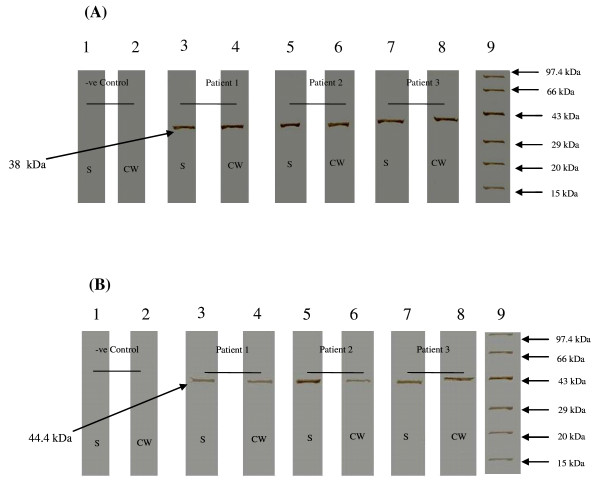
**Western blot assay for detection of anti-IncB and anti-IncC IgG antibodies**. Detection of (A) anti-IncB IgG and (B) anti-IncC IgG antibodies in sera (S) and cervical washes (CW) obtained from CT-positive patients and controls. Strips 3, 5, 7 – Detection of IncB IgG antibodies in sera (S) of patients' 1, 2, 3 respectively; strips 2, 4, 6- Detection of IncB IgG antibodies in cervical washes (CW) of patients' 1, 2, 3 respectively; strips 1, 2 – Serum and cervical wash respectively obtained from CT-negative patient (which served as negative controls for assay). Strip 9 – Molecular weight marker (Bangalore Genei). A 38 kDa band (IncB specific IgG) and 44.4 kDa band (IncC specific IgG) was detected in sera and cervical washes obtained from CT-positive patients. No bands were observed in negative control. IncB- CT Inclusion membrane protein B; IncC- CT Inclusion membrane protein C; CT- *Chlamydia trachomatis*; kDa- Kilodaltons; S- Serum; CW- Cervical washes.

Presence of IncB and IncC antibodies in cervical washes and sera was further confirmed by Western blot assay (Figure 2). Western blot assay showed presence of a 38 kDa band for IncB and a 44.4 kDa band for IncC in CT-positive samples. No corresponding bands were observed in cervical washes and sera obtained from controls.

### Cell proliferation

**Figure 3:**
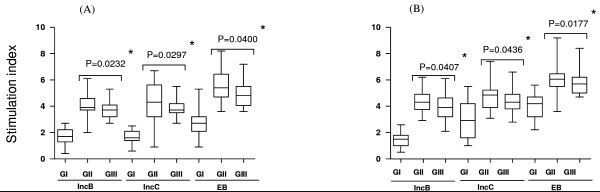
**MTT assay showing proliferative responses**. Proliferative responses (stimulation indices) of (A) cervical cells and (B) PBMCs isolated from GI, GII, GIII on stimulation with IncB, IncC and CT EB were estimated by MTT assay. (A) *Significant difference in proliferative responses in GII with respect to GIII (P = 0.0232, P = 0.0297 and P = 0.0400 upon stimulation with IncB, IncC and EB respectively). (B) *Significant difference in proliferative responses in GII with respect to GIII (P = 0.0407, P = 0.0436 and P = 0.0177 upon stimulation with IncB, IncC and CT EBs respectively) where; Group I (GI) comprised of healthy fertile women with no CT infection, Group II (GII) comprised of CT-positive fertile women, Group III (GIII) comprised of CT-positive women with fertility disorders. The horizontal line in the middle of the box is the median value of the responses and the lower (upper) is the 25th (75th) percentile., *Significant; PBMCs- Peripheral blood mononuclear cells; IncB- CT Inclusion membrane protein B; IncC- CT Inclusion membrane protein C; CT- *Chlamydia trachomatis*; EB- Elementary Bodies; n- Number. Proliferative responses between groups were evaluated using Mann-Whitney *U *test.

MTT assay was used to evaluate cellular proliferation of cervical cells and PBMCs upon stimulation with IncB and IncC. Proliferative responses (SI > 2) to IncB and IncC was statistically significant (P < 0·05) in cervical cells (Figure 3A) and PBMCs (Figure 3B) from CT-positive fertile women compared to CT-positive women with fertility disorders or controls. Stimulation with free GST (which served as negative controls) showed no significant differences in proliferative responses in any group (data not shown).

### Cell cytotoxity assays

Damage to plasma membranes of cells stimulated with incs was evaluated by measuring the release of LDH, a stable cytoplasmic enzyme into culture supernatants. No significant difference (P > 0.05) was observed in the number of positive responders for LDH release in cervical cells and PBMCs isolated from CT-positive patients and controls (Table 3). Free GST did not produce significant differences in LDH in any group (data not shown).

**Table 3:** Lactate dehydrogenase (LDH) assay for cell cytotoxicity

	**No. of patients' positive for LDH release from cells stimulated with chlamydial antigens**					
	**Cervical cells**			**PBMCs**		
**Groups**	**IncB**	**IncC**	**EBs**	**IncB**	**IncC**	**EBs**
	**n (%)**					
**GI**	2(6.45)	3(9.67)	2(6.45)	2(6.45)	2(6.45)	2(6.45)
**GII**	3(7.89)^a^	4(10.52)^b^	3(7.89)^c^	2(5.26)^d^	4(10.52)^e^	5(13.15)^f^
**GIII**	4(13.79)	3(10.34)	5(17.24)	4(13.79)	4(13.79)	3(10.34)

Note. Table shows the number of patients (n) positive for LDH release in culture supernatants of stimulated cells. Cervical cells and PBMCs were isolated from patients of GI, GII and GIII and stimulated with chlamydial antigens IncB, IncC and EBs. The number of patients positive for LDH release into culture supernatants post stimulation was noted. Values in parentheses represent corresponding percentages.

^a, b, c, d, e, f ^P not significant as compared to corresponding GI and GIII where, Group I (GI) comprised of healthy fertile women with no CT infection, Group II (GII) comprised of CT-positive fertile women, Group III (GIII) comprised of CT-positive women with fertility disorders.

LDH- Lactate dehydrogenase; PBMCs-Peripheral blood mononuclear cells; IncB- CT Inclusion membrane protein B; IncC- CT Inclusion membrane protein C; CT- *Chlamydia trachomatis*; EBs-Elementary bodies.

All categorical variables were compared using the χ^2 ^test.

### Cytokine mRNA expression levels in inc-stimulated cervical cells and PBMCs

**Figure 4:**
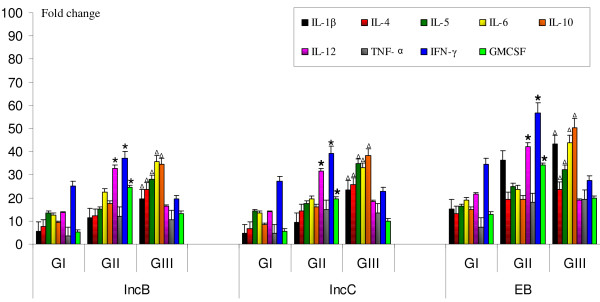
**Cytokine mRNA expression in inc-stimulated cervical cells**. Estimation of mRNA expression of IL-1β, IL-4, IL-5, IL-6, IL-10, IL-12, TNF-α, IFN-γ and GMCSF in 0.6 × 10^5 ^cervical cells after *in vitro *stimulation with IncB, IncC and CT EB. Real-time RT-PCR analysis of mRNA levels was done at 12 hours post infection in cervical cells isolated from GI, GII and GIII where, Group I (GI) comprised of healthy fertile women with no CT infection, Group II (GII) comprised of CT-positive fertile women, Group III (GIII) comprised of CT-positive women with fertility disorders. * P < 0.05 Expression of cytokine mRNA in GII compared to corresponding levels in GI and GIII by Kruskal Wallis test. ^Δ ^P < 0.05 Expression of cytokine mRNA in GIII compared to corresponding levels in GI and GII by Kruskal Wallis test. RT-PCR- Reverse-Transcriptase PCR; IncB- CT Inclusion membrane protein B; IncC- CT Inclusion membrane protein C; CT-*Chlamydia trachomatis*; EB-Elementary bodies; IL-Interleukin; β-Beta; IFN-γ-Interferon gamma; TNF-α-Tumor Necrosis factor alpha; GMCSF- Granulocyte macrophage colony-stimulating factor. All cytokines were normalised against corresponding levels of β-actin endogenous gene. The graph show results as fold change represented by bars. Bars represent mean ± s.e.m. for all experiments. X axis- Stimulants used in the study; Y-axis- Fold change in RNA expression under different conditions.

**Figure 5:**
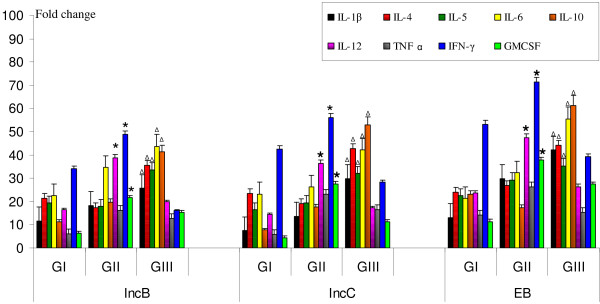
**Cytokine mRNA expression in inc-stimulated PBMCs**. Estimation of mRNA expression of IL-1β, IL-4, IL-5, IL-6, IL-10, IL-12, TNF-α, IFN-γ and GMCSF in 0.6 × 10^5 ^PBMCs after *in vitro *stimulation with IncB, IncC and CT EB. Real-time RT-PCR analysis of mRNA levels was done at 12 hours post infection in PBMCs isolated from GI, GII and GIII where, Group I (GI) comprised of healthy fertile women with no CT infection, Group II (GII) comprised of CT-positive fertile women, Group III (GIII) comprised of CT-positive women with fertility disorders. * P < 0.05 Expression of cytokine mRNA in GII compared to corresponding levels in GI and GIII by Kruskal Wallis test. ^Δ ^P < 0.05 Expression of cytokine mRNA in GIII compared to corresponding levels in GI and GII by Kruskal Wallis test. RT-PCR- Reverse-Transcriptase PCR; IncB- CT Inclusion membrane protein B; IncC- CT Inclusion membrane protein C; CT- *Chlamydia trachomatis*; EB-Elementary bodies; IL-Interleukin; β-Beta; IFN-γ-Interferon gamma; TNF-α-Tumor Necrosis factor alpha; GMCSF- Granulocyte macrophage colony-stimulating factor. All cytokines were normalised against corresponding levels of β-actin endogenous gene. The graph show results as fold change represented by bars. Bars represent mean ± s.e.m. for all experiments. X axis- Stimulants used in the study; Y-axis- Fold change in RNA expression under different conditions.

Messenger RNA for IL-1β, IL-4, IL-5, IL-6, IL-10, IL-12, TNF-α, IFN-γ and GMCSF was detected in stimulated cervical cells and PBMCs in all patients' groups. On stimulation of cervical cells (Figure 4) and PBMCs (Figure 5) with incs, significant increase in mRNA expression levels of IFN-γ, IL-12, and GM-CSF (P < 0.05) was observed in cervical cells and PBMCs obtained from CT-positive fertile women compared to other groups. In contrast, IL-1β, IL-4, IL-5, IL-6 and IL-10 mRNA expression levels were significantly higher (P < 0.05) in cells obtained from CT-positive women with fertility disorders compared to other two groups. There were no changes in cytokine mRNA expression in cervical cells and PBMCs stimulated with free GST (data not shown).

### ELISA for cytokines in cell supernatants of inc-stimulated cervical cells and PBMCs

Significantly higher levels of IL-1β, IL-6 and IL-10 were detected upon inc-stimulation of cervical cells (Table 4) and PBMCs (Table 5) from CT-positive women with fertility disorders as compared to CT-positive fertile women or controls (P < 0.05). In contrast, cervical cells and PBMCs obtained from CT-positive fertile women secreted significantly higher levels of IL-12, IFN-γ and GM-CSF compared to CT-positive women with fertility disorders or controls (P < 0.05). Significantly high levels of TNF-α and IL-6 levels were secreted in cervical cells and PBMCs from CT-positive fertile women compared to controls. IL-4 and IL-5 was below detection limit in all culture supernatants (data not shown). Cervical cells and PBMCs cultures stimulated with free GST showed no significant differences in levels of secreted cytokines (data not shown).

**Table 4:** Cytokine concentrations in culture supernatants of stimulated cervical cells

	**GI**			**GII**			**GIII**		
	**IncB**	**IncC**	**EBs**	**IncB**	**IncC**	**EBs**	**IncB**	**IncC**	**EBs**
**IL-1β**	**78** (49.5–235.9)	**93** (58.6–286.9)	**143.6** (52.1–362.76)	**143 **(61.8–381.5)	**167.2** (55.2–417.3)	**268** (48.6–473.6)	**293.2^a^** (77.5–731.4)	**327.5^b^** (65.3–821.8)	**521.1^c^** (86.2–921.1)
									
**IL-6**	**111** (38.2–371.5)	**127.4** (52.1–443.4)	**178.65** (67.9–871.76)	**398.4^d^** (82.8–671.8)	**431.56^e^** (97.6–544.9)	**631^f^** (111.7–931.6)	**732.3^a^** (241.3–1121.62)	**756.61^b^** (222.9–1006.8)	**1107.1^c^** (341–1621.8)
									
**IL-10**	**158.6** (100.1–267.2)	**172.2** (97.6–342.5)	**183.4** (92.6–512.46)	**366.9** (112.5–548.9)	**387.9** (126.1–561.9)	**537.8** (221–712.5)	**956.9^a^** (347.9–1231)	**869.7^b^** (358.7–1331.9)	**1259.8^c^** (568.7–1712.89)
									
**IL-12**	**152.5** (97.4–306.8)	**168.9** (88.8–277.1)	**251.8** (113.7–342.7)	**289.8^g^** (134.6–450)	**297.6^h^** (156.9–430.7)	**566.5^i^** (189.4–821.6)	**141.4** (68.9–281.8)	**156.8** (75.8–312.8)	**171.6** (126.9–400)
									
**IFN-γ**	**312.5^j^** (78.8–454.3)	**321.8^k^** (91.3–431.6)	**426.7^l^** (122.6–600.1)	**512.5^g^** (346.1–861.4)	**571.3^h^** (311.9–814.3)	**843.8^i^** (424.8–1272.5)	**168.2** (107.9–575.3)	**187.3** (142.7–612.3)	**313.6** (163.4–621.9)
									
**TNF-α**	**129.3** (43.6–212.5)	**136.9** (38.9–232.8)	**143.6** (54.1–312.6)	**256.3^d^** (115.7–385.4)	**234.6^e^** (114.8–412.6)	**411.7^f^** (134.2–532.7)	**226.4** (89.7–352.7)	**267.8** (97.3–411.9)	**320.4** (118.9–543.1)
									
**GM-CSF**	**116.1** (55.8–143.7)	**117.6** (48.3–129.8)	**135.2** (76.8–176.9)	**245.1^g^** (76.3–311.1)	**222.3^h^** (83.9–288.7)	**300.8^i^** (123.4–389.8)	**147.9** (63.6–176.4)	**132.4** (72.5–166.9)	**204.1** (89.2–237.1)

Note. Data represents median values (bold). Values in parentheses depict range. All values in pg/ml unless otherwise stated. Along the horizontal axis are the stimulants IncB, IncC and EBs used in cells obtained from GI, GII and GIII and vertical axis are the cytokines secreted in cell cultures assayed using specific ELISAs.

^a, b, c ^P < 0.05 Level of secreted cytokine in GIII compared to respective levels in groups I and II by Mann Whitney U test.

^d, e, f ^P < 0.01 Level of secreted cytokine in GII compared to respective levels in group I by Mann Whitney U test.

^g, h, i ^P < 0.05 Level of secreted cytokine in GII compared to respective levels in groups I and III by Mann Whitney U test.

^j, k, l ^P < 0.05 Level of secreted cytokine in GI compared to respected levels in group III by Mann Whitney U test where, Group I (GI) comprised of healthy fertile women with no CT infection, Group II (GII) comprised of CT-positive fertile women.

Group III (GIII) comprised of CT-positive women with fertility disorders. IncB- CT Inclusion membrane protein B; IncC- CT Inclusion membrane protein C; CT- *Chlamydia trachomatis*; EB-Elementary bodies; IL-Interleukin; β-Beta; IFN-γ-Interferon gamma; TNF-α-Tumor Necrosis factor alpha; GM-CSF- Granulocyte macrophage colony-stimulating factor

**Table 5:** Cytokine concentrations in culture supernatants of stimulated PBMCs

	**GI**			**GII**			**GIII**		
	**IncB**	**IncC**	**EBs**	**IncB**	**IncC**	**EBs**	**IncB**	**IncC**	**EBs**
**IL-1β**	**109.4** (56.7–258.3)	**119.7** (59.6–324.1)	**159.9** (73.1–462.69)	**172.6 **(68.4–393.8)	**183.2** (65.5–407.5)	**289** (73.1–491.4)	**413.36^a^** (82.1–786.3)	**432.5^b^** (95.1–729.8)	**568.9^c^** (76.4–821.7)
									
**IL-6**	**132.7** (54.3–457.9)	**146.3** (67.21–547.1)	**201.65** (87.9–719.82)	**487.6^d^** (88.86–791.1)	**473.58^e^** (98.6–564.7)	**668^f^** (121.4–956.8)	**813.6^a^** (300.2–1421.4)	**793.45^b^** (312.9–1201.4)	**1335.47^c^** (436.1–1578.3)
									
**IL-10**	**183.4** (106.3–383.6)	**196.3** (87.3–446.3)	**328.4** (88.1–612.46)	**462.7** (189.2–631.3)	**411.21** (166.8–661.7)	**554.9** (246–745.2)	**1005^a^** (414.2–1426.3)	**956.2^b^** (437.7–1431.6)	**1472.8^c^** (336.2–1803)
									
**IL-12**	**204.8** (113.7–342.7)	**198.7** (96.8–327.1)	**342.7** (234.7–515.1)	**466.5^g^** (173.9–712.6)	**450.1^h^** (167.2–572.8)	**621.1^i^** (188.3–745.8)	**167.4** (75.8–312.8)	**156.8** (88.3–345.8)	**186.36** (129.3–621.3)
									
**IFN-γ**	**453.7^j^** (88.5–549.3)	**511.3^k^** (87.3–631.6)	**578.8^l^** (311.9–714.3)	**726.6^g^** (351.3–1128.4)	**671.3^h^** (454.8–1272.5)	**884.7^i^** (557.4–1377.2)	**241.7** (142.-612.3)	**196.8** (157.6–661.7)	**263.6** (163.4–721.9)
									
**TNF-α**	**143.6** (54.1–312.6)	**156.3** (48.9–331.5)	**264.2** (127.4–423.7)	**314.7^d^** (119.7–523.8)	**293.6^e^** (127.4–623.7)	**311.7^f^** (113–452.9)	**289.9** (97.3–411.9)	**277.2** (113–452.9)	**289.3** (125.6–543.7)
									
**GM-CSF**	**128.7** (63.4–156.8)	**136.4** (71.3–149.4)	**152.4** (89.4–201.1)	**263.1^g^** (125.3–291.2)	**248.1^h^** (111–321.2)	**320.4^i^** (136–520)	**151.2** (76.1–184)	**163.4** (91.3–201)	**268.7** (100.1–306.9)

Note. Data represents median values (bold). Values in parentheses depict range. All values in pg/ml unless otherwise stated. Along the horizontal axis are the stimulants IncB, IncC and EBs used in cells obtained from GI, GII and GIII and vertical axis are the cytokines secreted in cell cultures assayed using specific ELISAs.

^a, b, c ^P < 0.05 Level of secreted cytokine in GIII compared to respective levels in groups I and II by Mann Whitney U test.

^d, e, f ^P < 0.01 Level of secreted cytokine in GII compared to respective levels in group I by Mann Whitney U test.

^g, h, i ^P < 0.05 Level of secreted cytokine in GII compared to respective levels in groups I and III by Mann Whitney U test.

^j, k, l ^P < 0.05 Level of secreted cytokine in GI compared to respected levels in group III by Mann Whitney U test where, Group I (GI) comprised of healthy fertile women with no CT infection, Group II (GII) comprised of CT-positive fertile women.

Group III (GIII) comprised of CT-positive women with fertility disorders. PBMCs-Peripheral blood mononuclear cells; IncB- CT Inclusion membrane protein B; IncC- CT Inclusion membrane protein C; CT- *Chlamydia trachomatis*; EBs-Elementary bodies; IL-Interleukin; β-Beta; IFN-γ-Interferon gamma; TNF-α-Tumor Necrosis factor alpha; GM-CSF- Granulocyte macrophage colony-stimulating factor.

### Antigen specific CD4^+ ^T cell responses

IL-4 and IFN-γ spot forming cells (SFCs) were enumerated in inc-stimulated CD4^+ ^T cells purified from cervical cells and PBMCs in CT-positive women with and without fertility disorders and controls. As shown in Table 6, CD4^+ ^T cells in CT-positive fertile women exhibited significantly elevated numbers of IFN-γ SFCs, in a dose-dependent fashion, upon stimulation with 0.5 μg/ml or 1 μg/ml of both IncB and IncC, as compared to cells from CT-positive women with fertility disorders. Cells stimulated with EBs (positive control) showed maximal IFN-γ SFCs whereas cells stimulated with free GST (negative control) showed negligible IFN-γ production (data not shown). Further, frequency of IFN-γ secreting cells was highest among CD4^+ ^T cells in cervical cells and PBMCs obtained from CT-positive fertile women followed by controls. In CT-positive women with fertility disorders, significantly (P < 0.05) lower number of IFN-γ SFCs was present. There was no detectable IL-4 SFCs in any of the cell cultures (data not shown).

**Table 6:** IFN-γ ELISPOT assay in IncB or IncC stimulated CD4^+ ^T cell cultures

	**GI**				**GII**				**GIII**			
	**IncB (μg/ml)**		**IncC (μg/ml)**		**IncB (μg/ml)**		**IncC (μg/ml)**		**IncB (μg/ml)**		**IncC (μg/ml)**	
	0.5	1	0.5	1	0.5	1	0.5	1	0.5	1	0.5	1
***Cervical cells***	**111** (48–214	**126** (67–263)	**91** (29–167)	**142** (43–211)	**132^a^** (47–312)	**157^b^** (56–343)	**112^c^** (38–112)	**178^d^** (77–329)	**43** (12–59)	**49** (12–88)	**38** (17–63)	**59** (26–76)
												
***PBMCs***	**121** (38–200)	**134** (45–212)	**103** (48–176)	**172** (44–176)	**212^a^** (56–273)	**226^b^** (76–301)	**243^c^** (91–322)	**251^d^** (100–316)	**56** (38–97)	**67** (43–115)	**63** (59–108)	**81** (60–149)

Note. Median number of IFN-γ SFCs per 7.5 × 10^4 ^CD4^+ ^T cells at 18 h post stimulation with IncB or IncC (0.5 μg/ml and 1 μg/ml) counted using ELISPOT. Figures in parenthesis depict range of SFCs for each group.

^a, b, c, d ^P < 0.05 Number of IFN-γ in GII compared to respective levels in group III by Mann Whitney U test where, Group I (GI) comprised of healthy fertile women with no CT infection, Group II (GII) comprised of CT-positive fertile women.

Group III (GIII) comprised of CT-positive women with fertility disorders.

IncB- CT Inclusion membrane protein B; IncC- CT Inclusion membrane protein C; CT- *Chlamydia trachomatis*; EB-Elementary bodies; IL-Interleukin; IFN-γ-Interferon gamma; SFCs-Spot forming cells.

**Figure 6:**
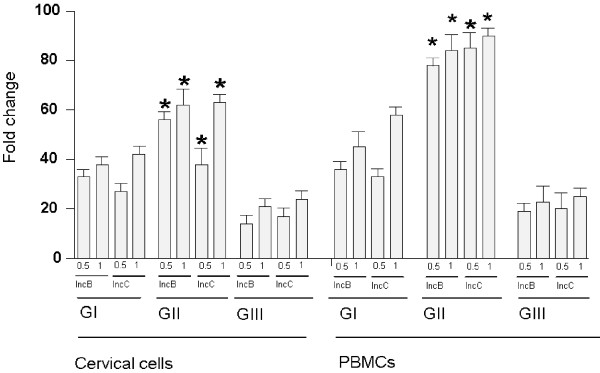
**Estimation of IFN-γ mRNA relative expression in 7.5 × 10^4 ^CD4^+ ^T cells isolated from cervical cells and PBMCs after *in vitro *stimulation with IncB or IncC**. Real-time RT-PCR analysis of IFN-γ mRNA levels was done at 12 hours post stimulation in CD4^+ ^T cells isolated cervical cells and PBMCs isolated from GI, GII and GIII where, Group I (GI) comprised of healthy fertile women with no CT infection, Group II (GII) comprised of CT-positive fertile women, Group III (GIII) comprised of CT-positive women with fertility disorders. * P < 0.05 mRNA expression of IFN-γ in GII compared to respective levels in group III by Mann Whitney U test. All cytokines were normalised against corresponding levels of β-actin endogenous gene. The graph show results as fold change represented by bars. Bars represent mean ± s.e.m. for all experiments.

Real time RT-PCR analysis showed significant (P < 0.05) increase in IFN-γ mRNA in purified CD4^+ ^T cells obtained from cervical cells and PBMCs in CT-positive fertile women compared to CT-positive women with fertility disorders (Figure 6). Relative expression of IFN-γ was higher in CT-positive fertile women and controls with respect to CT-positive women with fertility disorders. Expression of IL-4 was low and not significant in CT-positive women and controls (data not shown).

## Discussion

To the best of our knowledge, this is the first study on mucosal and peripheral immune responses to chlamydial inclusion proteins in CT-positive women with or without fertility disorders. Although several inc proteins have been identified in different chlamydial species however, their role in chlamydial biology, especially with respect to generating host immune responses in not clear. Being present at the cytoplasmic face of the inclusion, incs are critical for direct interactions with proteins in the host cell cytosol [11,12,42,43].

Using reported [35], and unique primer sets, we were able to detect *incB *and *incC *genes in CT-positive endocervical samples. The prevalence of antibodies against IncB and IncC in cervical washes was detected significantly high in CT-positive fertile women compared to CT-positive women with fertility disorders showing their involvement in initial infection than in established disease pathology. However, antibodies to IncB and IncC were not significantly different in sera in the same patients' groups. The differential immunogenic properties of inclusion proteins and their involvement in particular disease pathologies may be attributed to the native conformational structures of proteins *in vivo *and their cellular localization. Further, new information on the 3 dimensional structures of IncB and IncC is required which will be helpful in understanding arrangement of epitopes necessary for generating antibodies against these proteins within infected host cells. High prevalence of antibodies against CT MOMP antigen in patients with primary chlamydial infections has also been reported [38]. In addition to their roles in neutralizing infectious organisms, antibodies to CT surface elements are known to dramatically enhance opsonization of dendritic cells and to promote strong Th1 immune responses [44]. Conversely, antibodies against cHSP60 antigen in patients with recurrent infections [38] and against recombinant cHSP60 proteins in women with fertility disorders have also been reported [45]. Serological data have shown a strong association between antibody responses to cHSP60 and sequalae of CT infection, including PID, tubal infertility, ectopic pregnancy, and scarring trachoma [46-50].

Cellular proliferation in cervical cells and PBMCs using MTT assay showed higher stimulation indices for IncB or IncC in CT-positive fertile women as compared to controls and CT-positive women with fertility disorders. Previous studies on lymphocyte proliferation to chlamydial antigens, including MOMP and heat shock protein 60, have shown enhanced responses in individuals who spontaneously resolve trachoma infection as compared to those with persistent infections [51]. In another study relative depression in lymphocyte proliferation in response to CT antigens in subjects with persistent clinical signs of inflammatory trachoma and in subjects with severe trachomatous scarring has been reported too [52]. CT IncC has been reported to have insignificant effect on altering host cell cytokinesis whereas CT 223–227 caused maximum reduction in host cell cytokinesis and facilitated centromere supranumeracy defects in transfected HeLa or McCoy cells [53]. Further our data on the number of positive responders for LDH release showed no significant differences between CT-positive patients and controls. Thus both incs failed to cause any disruption of cellular integrity of stimulated cervical cells and PBMCs thereby suggesting that these incs could initiate specific cellular immune responses.

Results in this study suggest that exposure to incs could significantly affect host immune function by modifying the release of cytokines. On comparing cytokine expression by cells obtained from CT-positive fertile women with those with controls and CT-positive women with fertility disorders, cells from CT-positive women with fertility disorders secreted significantly higher levels of IL-1β. The probable role of IL-1 in fallopian tube destruction during CT infection has been reported [54]. Further, it is also shown that synovial tissues from chronic arthritis patients with synovial *C. pneumoniae *infection have significant levels of mRNA for IL-1β [55]. These results collectively confirm that IL-1β can be one of the cytokines that are responsible for chlamydial pathology.

Both incs induced high levels of IL-6 in cells from CT-positive fertile women with or without fertility disorders in comparison to controls. In CT-infected cells, IL-6 has been reported to act synergistically with IL-12, for inducing protective immune responses [56]. However, our data showed that highest IL-6 levels were produced in inc-stimulated cells from CT-positive patients with fertility disorders suggesting the pathogenic role of IL-6. High levels of IL-6 levels have been previously reported to be present in the tubal fluids of patients with infertility caused by chlamydial infections [57]. In another report, high IL-6 levels have been found to be secreted from CT EB-stimulated cervical cells obtained from CT-positive infertile women [58].

IL-10 was found to be up-regulated in both cervical cells and PBMCs stimulated with incs in CT-positive women with fertility disorders, with levels being significantly higher than CT-positive fertile women and controls. In a previous report on a longitudinal study of genital CT infection, *in vitro *IL-10 production by PBMCs stimulated with recombinant chlamydial antigens was the greatest risk factor for recurrent infections [59]. In murine models of chlamydial infection, high levels of IL-10 impede pathogen clearance [60], whereas IL-10 mutant mice clear infection faster than genetically intact controls [61-63]. IL-10 has been found to be associated with susceptibility to chlamydial infection and typical pathological changes caused by the infection such as granuloma formation and fibrosis [64]. Excessive levels of IL10 levels in conjunctiva are a risk factor for scarring and blindness during CT-associated trachoma. IL-10 contributes to the pathogenesis of fibrotic responses [61,65], and is involved in local matrix remodelling during healing and repair of inflammation-induced injury [66]. Similarly our data suggests that IL-10 over-expression in cells from CT-positive women with fertility disorders could partly contribute to slow or non-clearance of CT resulting in establishment of tubal pathology.

Levels of IFN-γ were significantly high in inc-stimulated cells from CT-positive fertile women whereas in CT-positive women with fertility disorders IFN-γ was found to be downregulated. Debattista *et al*., have reported that women with chlamydial PID or a history of repeated CT infection had PBMCs that produced less IFN-γ in response to CHSP60 than women with a single episode of CT infection [67]. Subjects with scarring trachoma have shown to produce reduced IFN-γ levels during persistent chlamydial infection [52]. Also, co-occurrence of IL-10 and IFN-γ producing cells in synovial tissues from CT-positive arthritis patients have suggested that excessive IL-10 production suppresses IFN-γ and mediates persistence [68]. These results along with ours suggest a protective role of IFN-γ during active chlamydial infection.

Further we detected levels of IL-12 to be significantly higher in inc-stimulated cervical cells and PBMCs from CT-positive fertile women compared to controls and CT-positive women with fertility disorders. IL-12 is primarily produced by activated macrophages and dendritic cells and further induces IFN-γ production to favour the differentiation of Th1 cells [69,70]. Along with TNF-α, IL-12 is reported to co-stimulate IFN-γ by killer T cells [71]. It has also been previously reported that clearance of chlamydial infection from female adolescents has been associated with decrease in IL-12 concentrations in endocervical samples suggesting its role in protective immune responses against infection [72].

Levels of TNF-α were significantly higher in inc-stimulated cervical cells and PBMCs from CT-positive women with or without fertility related disorders compared to controls. TNF-α is known to inhibit chlamydial growth *in vitro *[73] and *in vivo *[74,75]. Further, along with IFN-γ, TNF-α is reported to have implications in infertility [76,77].

Levels of GM-CSF were significantly higher in CT-positive fertile women compared to CT-positive women with fertility disorders and controls. GM-CSF is known to activate macrophages and up-regulate CD14 and MHC class II expression [78], suggesting its role in protective immunity. Further, in a murine model of CT mouse pneumonitis lung infection and intrapulmonary adenoviral GM-CSF transfection, it has been demonstrated that the expression of GM-CSF in the airway compartment significantly enhanced systemic Th1 cellular and local IgA immune responses following immunization with inactivated organisms [79].

A significant finding of this study was the increased mRNA expression levels of IL-4, a classical Th2 marker, in cells obtained from CT-positive women with fertility disorders in comparison to the other two groups. It has previously been reported that individuals with severe trachomatous scarring had PBMCs that produced IL-4 to CHSP60 more frequently than did PBMCs from matched community control subjects without scarring [52]. We failed to detect IL-4 protein levels by ELISA which may be due to the fact IL-4 secreting cells may be quenched or short lived in peripheral circulation or its secretion was downregulated by other factors [52]. Depressed levels of IL-4 has also been detected in CT-infected HIV positive patients using TaqMan™ based real time PCR [80]. In addition in this study, IL-5 mRNA, another Th2 cytokine was also elevated in the same cell population. Elevated levels of IL-4 and IL-5 by splenic lymphocytes from IFN-γ knock-out mice following chlamydia-specific has been reported. It was observed that this Th2 mediated delayed hypersensitivity was not protective in controlling local chlamydial infection and associated with serious tissue damage and cellular inflammation and failing to prevent dissemination of the pathogen to other organs in these mice [81].

Overall our data on cytokine modulation in cervical cells and PBMCs on *in vitro *stimulation with IncB or IncC suggests that CT- negative women who encounter chlamydial antigens for the first time and CT-positive fertile women produce higher amounts of IFN-γ, IL-12 and GM-CSF than in women with reproductive sequalae to chlamydial infection where the infected cells secrete higher levels of IL-1β, IL-4, IL-5, IL-6 and IL-10.

In addition CD4^+ ^T cells in CT-positive women with and without fertility disorders displayed high levels of IFN-γ secreting cells and mRNA expression and minimal IL-4 production upon *in vitro *dose-dependent stimulation with IncB and IncC, demonstrating the induction of an antigen-specific Th1 cellular response. There is accumulated evidence to suggest that Th1 responses and IFN-γ production are important for optimal resolution of genital chlamydial infection [2,3]. It has also been demonstrated that *Chlamydia-*specific Th1 clones, but not Th2 clones, are capable of adoptively transferring anti-chlamydial immunity [82-84], and that MHC class II deficient, but not MHC class I deficient animals were incapable of resolving a primary chlamydial challenge [85]. Thus, our data shows that IncB and IncC could be able to mount a protective Th1 cellular response for clearance of CT from infected host cells. Although it would be presumptuous to assume that the clinical condition of women with fertility disorders could be completely attributed to chlamydial infections yet it is not wrong to say that the changes in cytokine patterns can be responsible for development of various pathological consequences to chlamydial infection. Further, to this end the role of IncB and IncC in maintaining a differential cytokine surveillance milieu within infected cells is of interest. As chlamydial inclusion proteins are involved in biochemical cross-talk between host cells and infecting pathogens, hence the overall data on host immune responses to these proteins would be helpful in further understanding of biological functioning of this elusive pathogen.

## Conclusion

The intent of the study was to provide insights into the role of chlamydial inclusion membrane proteins in generating immunity in host cells and their probable contribution to the disease processes associated with chlamydial infections. This study suggested that the chlamydial IncB and IncC are able to generate differential humoral and cell mediated immune responses in *Chlamydia*- infected women with or without fertility disorders. Further research is warranted to precisely define the potential contribution of chlamydial incs in immunopathology associated with chlamydial infection.

## Competing interests

The authors declare that they have no competing interests.

## Authors' contributions

AM and RG had participated in the design of the study. The experiments were carried out by RG. PS and HV had assisted in Western blotting experiments. Data analysis was performed by RG. SS and PS had helped in collection of samples. AM and RG drafted and wrote the manuscript. All authors have read and approved the final manuscript.
